# The impact of a group music game intervention on cooperative behavior in 5- to 6-year-old children

**DOI:** 10.3389/fpsyg.2026.1763858

**Published:** 2026-01-29

**Authors:** Xi Yang, Rong Peng, Yutong He, Fangfei Li, Zuokun Qin, Shiqi Lei, Dan Kang

**Affiliations:** 1 Key Laboratory of Cognition and Human Behavior in Hunan Province, School of Educational Science, Hunan Normal University, Changsha, China; 2 Kindergarten Affiliated to Hunan Normal University, Changsha, Hunan, China

**Keywords:** child development, cooperative behavior, group music games, interpersonal synchrony, staged intervention

## Abstract

This study examined the impact of an 8-week group music game intervention on cooperative behavior in 5- to 6-year-old children. A 2 × 3 mixed experimental design was employed, with 60 children randomly assigned to either an experimental group (*n* = 30) or a control group (*n* = 30). The intervention consisted of movement-synchronized rhythm games during the first 4 weeks, followed by instrumental ensemble games in the final 4 weeks. Cooperative behavior was assessed using the Truck Racing Task before, during, and after the intervention. Repeated-measures ANOVA revealed that: (1) the rhythm game phase significantly improved cooperative behavior; (2) the instrumental ensemble phase produced further additive gains; and (3) intervention effects remained stable after a one-week follow-up, indicating short-term sustainability. These findings demonstrate for the first time that structured group music games can effectively enhance cooperative behavior in naturalistic classroom settings, offering empirical support for the use of music-based interventions in early social development.

## Introduction

Cooperation refers to coordinated social interaction among multiple individuals to achieve a common goal (Hay et al., 2022; Tomasello et al., 2005) and is a core competency in children’s social development. The preschool period is a critical stage for the development of cooperative abilities. The cooperative abilities formed during this stage not only lay the foundation for children to establish positive peer relationships but also have a profound impact on their cognitive development and social adaptation (Tang et al., 2025; Yuill et al., 2014; Etel and Slaughter, 2019).

The development of early cooperative behavior exhibits a distinct age-graded pattern. Research indicates that infants can perceive others’ goals and exhibit a tendency to help as early as their first year of life (Slocombe and Seed, 2019), and by 12 months of age, they begin to develop coordinated interactions with peers (Fantasia et al., 2014; Kaartinen et al., 2019). As children grow older, their cooperative behavior evolves from simple dyadic interactions to complex group collaboration (Fitzgerald and Frankie, 1982), with significant improvements in the purposefulness and stability of their behavior (Warneken et al., 2006; Gräfenhain et al., 2013). This developmental trajectory is shaped by both environmental and individual factors.

Among various influencing factors, interpersonal synchrony has been widely supported as a key mechanism promoting cooperative behavior (Wiltermuth and Heath, 2009; Rennung and Göritz, 2016; Stupacher et al., 2017; Cirelli, 2018; daSilva and Wood, 2025; Tarr et al., 2014; Rabinowitch and Meltzoff, 2017). Interpersonal synchrony refers to the coordinated alignment of behavior across time dimensions among two or more individuals (Cirelli, 2018). It encompasses not only motor synchronization of behavioral actions but also temporal synchronization of physiological activities or emotional processes (Lin et al., 2024; Mayo and Gordon, 2020). Existing research suggests that interpersonal synchrony may enhance joint attention abilities (Tomasello et al., 2005), induce positive emotional experiences (Galbusera et al., 2019; Kim, 2025), reducing psychological distance to enhance prosocial tendencies (Cirelli et al., 2014), influencing the brain’s reward system (Wang et al., 2021), and activating the endogenous opioid system (Loseth et al., 2014; Mogan et al., 2017) among other pathways, to promote cooperative behavior.

Music activities, due to their inherent rhythmic characteristics, are considered an ideal medium for interpersonal synchronization (Tarr, 2017; Lang et al., 2016; Demos et al., 2012; Wiltermuth and Heath, 2009). Group music games are particularly valuable for research due to their rhythmic, interactive, and emotionally evocative characteristics (Beck and Rieser, 2020), which can effectively activate the collective intentionality of “synchronized vocalization and movement” (Kirschner and Tomasello, 2010). However, there remains disagreement regarding the impact of musical synchrony on cooperative behavior: supportive evidence suggests that, under laboratory conditions, 4-year-old children exhibit significantly enhanced prosocial intentions following synchronized musical activities (including singing, dancing, and playing musical instruments together) (Kirschner and Tomasello, 2010). However, Baier et al. (2021) conducted a field experiment in a kindergarten setting and found no significant differences in prosocial behavior between the music condition and the non-music condition, with no effects of gender or age, suggesting that the original results may have been overestimated and emphasizing that differences between laboratory and field experiments may lead to inconsistent results.

The above discrepancies may be due to the following reasons: first, both studies were single-session short-term interventions, which may have led to unstable results. Additionally, the laboratory environment may have amplified the effects of the music intervention, while the real-world environment allowed children to respond more naturally to music activities, thereby weakening the effects. Second, Kirschner and Tomasello (2010) incorporated a multi-dimensional collaborative approach to music intervention, including singing, dancing, and instruments, whereas Baier et al. (2021) separated these conditions, potentially reducing collaborative effects. Finally, both studies assessed prosocial behavior immediately after a single intervention, without considering the delayed effects or cumulative effects of the intervention.

Based on this, the present study innovatively implemented an 8-week phased group music game intervention program (the first 4 weeks focused on rhythm games emphasizing movement synchronization, followed by 4 weeks of instrumental ensemble games using musical instruments) to systematically examine the short-term sustained promotional effects of music synchronization activities on cooperative behavior in 5- to 6-year-old children in a kindergarten classroom setting. The study design has the following prominent features: (1) Long-term intervention in a natural educational setting addresses the methodological limitations of previous single-session laboratory studies, which lacked ecological validity, and short-term on-site interventions, which showed insignificant effects; (2) Through a progressive design from rhythmic synchronization to instrumental ensemble, the study verifies the sequential synergistic effects of different types of music synchronization activities on cooperative behavior; (3) A dynamic tracking model of “intervention-interval-assessment” is adopted, with assessments conducted 1 week after the conclusion of each intervention phase. This approach avoids the practice effects of immediate assessments while also testing the delayed stability of intervention effects.

## Materials and methods

### Aims

This study employed a 2 × 3 mixed experimental design (Group: experimental/control × Time: T1/T2/T3) to examine the effects of an 8-week, two-phase music intervention on cooperative behavior in 5–6-year-olds. The experimental group received 16 sessions (twice weekly), with the first 4 weeks focusing on movement-synchronized rhythm games and the latter 4 weeks on instrumental ensemble games, while the control group continued regular activities. We hypothesized that:

1. Rhythmic games based on movement synchronization (weeks 1–4) would significantly improve cooperative behavior;
2. Instrumental ensemble games (weeks 5–8) would produce additive gains beyond rhythm training;
3. Intervention effects would remain stable in delayed assessments, with the experimental group outperforming controls.

### Participants

*A priori* power analysis was conducted using G*Power 3.1 (Faul et al., 2007) for a 2 × 3 mixed-design ANOVA (between-factor: group; within-factor: time). With an anticipated large effect size (*f* = 0.425, equivalent to Cohen’s *d* = 0.85 based on prior music intervention studies), α = 0.05 (two-tailed), power = 0.85, and an assumed repeated-measures correlation of *ρ* = 0.5, the analysis indicated a minimum requirement of 26 participants per group. To accommodate potential attrition and enhance statistical robustness, we recruited 60 typically developing children (27 boys, 33 girls; mean age = 5.6 ± 0.3 years) from kindergarten senior classes, with 30 participants randomly assigned to each group. The experimental group comprised 16 girls and 14 boys, while the control group consisted of 17 girls and 13 boys.

Inclusion criteria were: (1) Middle socioeconomic status (SES) families; (2) No prior participation in structured prosocial or music-based interventions; (3) No developmental disabilities or cognitive impairments (verified through kindergarten health records).

Randomization was performed by an independent researcher using computer-generated random numbers (block randomization with a 1:1 allocation ratio), with balanced gender ratios (experimental: control = 7:8).

### Procedure

#### Ethical review

All studies involving participants were conducted with the consent of teachers and parents. The research content and process were approved by the ethics committee of the authors’ university, and the parents of the research participants all signed informed consent forms.

#### Research phases

This study was divided into a pre-experimental phase and a formal experimental phase. Two weeks before the formal experiment, six boys and six girls were selected for the pre-experiment to validate the appropriateness of the intervention program. Subsequently, the formal experimental phase began, with the experimental group of preschoolers undergoing an eight-week group music game intervention. The first 4 weeks focused on rhythmic music games emphasizing movement synchronization, while the final 4 weeks involved more complex instrumental ensemble game interventions. One week before the formal experiment began, pre-tests of cooperative skills were conducted for both the experimental and control groups. To avoid practice effects, mid-tests and post-tests were conducted 1 week after the intervention, specifically the mid-test of cooperative skills was conducted before the fifth week of intervention, and the post-test was conducted in the ninth week.

During the 8-week intervention period, the control group continued their regular kindergarten curriculum (including free play, arts, and storytime activities) without any structured music-based cooperation training.

### Materials and instruments

#### Group music games

The intervention consists of 16 group music game activities, with the first 8 activities focusing on rhythmic games emphasizing movement synchronization, and the latter 8 activities involving more complex instrumental ensemble games.

The intervention program was developed through collaborative discussions among university faculty members specializing in early childhood music education, graduate students, and kindergarten teachers. Feedback was solicited via email from 3 university faculty members and 5 kindergarten teachers specializing in early childhood education, and the program content was revised based on their written suggestions. Additionally, a pre-experiment was conducted with 12 randomly selected non-experimental group preschoolers in kindergarten prior to the intervention, focusing on observing children’s understanding of cooperative tasks, such as their ability to independently negotiate instrument allocation, the appeal of musical materials, and the effectiveness of cooperative behavior triggers. The intervention plan was adjusted based on the pre-experiment results and finalized (specific plan excerpts are shown in Table 1).

**Table 1:** Intervention plan (partial).

Activity type	Activity name	Activity content	Activity objectives
Rhythm game	The rhythm magic of lollipops	Play “Lolipop” and guide the children to perform individual, pair, and group movements in sequence. Finally, let them form teams freely to participate in a group dance and complete the rhythm challenge	Feel the joy of being part of a group through group interaction and stimulate enthusiasm for participation Improve the ability to coordinate personal and group rhythms and interact with others Understand the impact of personal rhythms on the group and the whole
	Please dance with me	Warm up with “A Ram Sam Sam” and guide the children to perform individual, group, and pair movements, exchanging dance partners during the interaction	Experience the joy of group games in group reorganization and enhance the willingness to interact with peers Improve the ability to adapt to new peer interactions and respond to changes in group size Perceive the effect of changes in group size on cooperation
	Passing game	Listen to and identify changes in the rhythm of musical passages, and understand the relationship between actions such as “passing the cup” and “clapping hands” and the rhythm. Work with peers to complete group and whole-class passing exercises in time with the rhythm	Feel a sense of accomplishment through precise coordination in rhythmic interaction, stimulating interest in participation Improve the ability to observe peers and synchronize rhythmic movements Clarify the relationship between movements and musical rhythms
Instrumental concerto game	Traffic rhythm battle	Using sound effects such as car horns and train whistles combined with dynamic music, young children hold homemade props such as “steering wheels” and “railway track models.” They use instruments (drums to simulate engines and cymbals to simulate brakes) to complete the “rhythm of urban traffic”	Experience the sense of accomplishment that comes with playing instruments together and enhance interest in collaboration Improve rhythm synchronization skills when playing drums and cymbals in pairs and groups Master rhythm patterns and connections between drums and cymbals in different contexts
	How to wake up the fat piggy	Create a scenario for waking up the piglets. Use bells and double-sided drums to set different “wake-up rhythms” for each group. Each group plays their rhythm, and eventually everyone plays together in sync to “wake up the piglets”	Experience the sense of accomplishment that comes from working together to achieve a goal, and inspire emotional collaboration through playing music Improve the ability to play in unison within a group and to collaborate across groups Understand the logic behind the coordination of rhythms between different groups of instruments
	Frozen rhythm	Play a variation of “Let It Go” and divide different types of instruments (wooden, iron, and resonant) into groups to perform together, creating the musical “Ice and Snow Kingdom Celebration”	Experience the joy of collective music creation and enhance participation Achieve precise rhythmic coordination among different instrument groups Grasp the collaborative mechanism of rhythmic synchronization in collective performance

This study conducted interventions in the form of group music games in the classroom activity room, with each session lasting 25–30 min, twice a week, for a total of 16 sessions over 8 weeks. Each activity was divided into four stages, with the specific process as follows:

1. Contextual Introduction: Through the creation of a story-based scenario, a “cooperation gap” is established to foster a group atmosphere. The core task of “collaborative activation” is anchored to stimulate children’s interest in participation, establishing a connection between “individual participation” and “collective goals” to drive the generation of cooperative intentions, ensuring children understand the need to work together.
2. Imitation Experience: Teachers guide children to actively participate in music games, using skill imitation through body movement and instrument operation to simultaneously instill “follow-instructions collaboration” and “spatial coordination” rules. This process combines “motor skill acquisition + social rule internalization” to help children understand “how to do it.”
3. Group Collaboration: Using “rhythm task division” as a medium, children voluntarily or randomly form different groups, each assigned specific tasks (such as actions or instrument playing with different rhythms), to directly experience the essence of collaboration where “tasks differ but goals are aligned,” enabling children to “do their part well.”
4. Comprehensive application: Through synchronized demonstrations of movements with different rhythms and collaborative performances of various instruments with different rhythms, while occasionally introducing small challenges (such as “suddenly increasing the music speed” or “adding a new movement”), children experience the process of “individual or group efforts” converging into “collective achievements,” feeling the sense of accomplishment that “collective effort is more powerful than individual action,” and learning to “adjust for a common goal.”

#### Truck racing task

We assessed cooperative behavior using the validated Truck Racing Task (Deutsch, 1973), which has demonstrated reliability in preschool studies (Nalis et al., 2018; Shomer et al., 1966). To ensure scoring objectivity, two trained early childhood education graduate students independently rated all cooperative behavior assessments, and the raters were blind to the children’s group assignments, which significantly strengthens the internal validity. Raters completed standardized training comprising: (1) a detailed review of scoring protocols, (2) consensus calibration through video analysis, and (3) practice coding achieving excellent reliability (*κ* > 0.85). Final inter-rater reliability remained high (*κ* = 0.92), with discrepancies resolved through joint review by the first author and corresponding author. Periodic reliability checks (10% random resampling) confirmed ongoing scoring consistency (*κ* > 0.90 throughout data collection).

**Figure 1:**
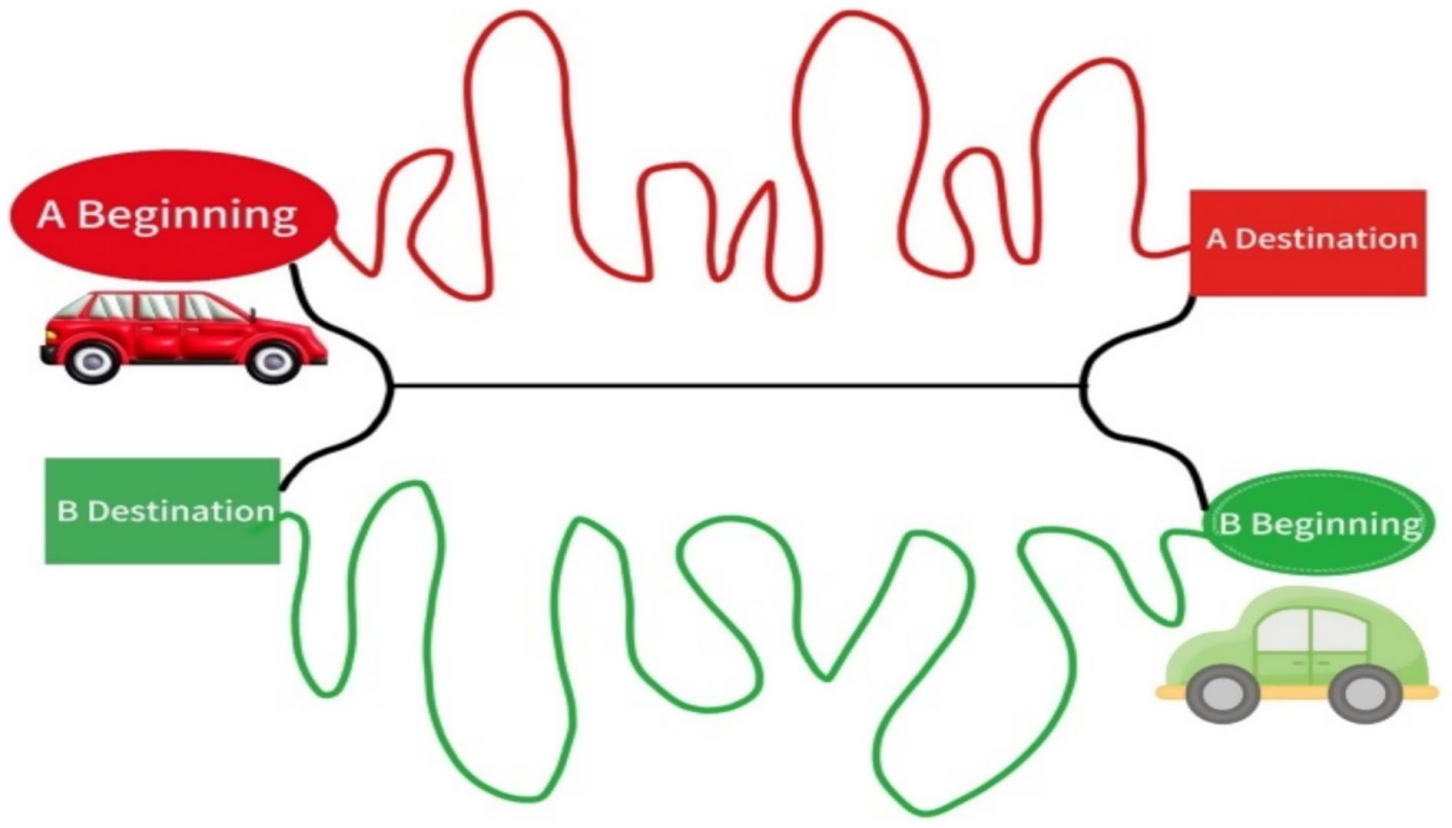
Truck racing task.

The measurement tool is used as follows: Children participate in pairs, each driving Truck A or Truck B, following a route map (as shown in Figure 1). Each truck is required to depart from the starting point and reach the destination as quickly as possible (time is not actually measured; only the children’s path selection is observed). Each truck has its own dedicated route, but this route is long and time-consuming. A one-way road is set up in the middle, which both parties can use but only one truck at a time. This one-way road is short, and the waiting time increased by alternating use of the one-way road is more economical and efficient than using the backup route. Communication between the two parties is allowed during the experiment. Finally, the level of cooperative behavior is scored based on the routes chosen by the two children.

Level 0: If the children cannot complete the task due to mutual opposition, they receive 0 points. Level 1: If both children take the side path to reach their respective destinations, they receive 1 point. Level 2: If one child takes the side path and the other takes the middle one-way path to reach their respective destinations, they receive 2 points. Level 3: If both children take turns using the middle one-way path to reach their respective destinations, they receive 3 points. The higher the score, the higher the level of cooperative behavior.

## Results

### Baseline equivalence

Independent samples *t*-test confirmed baseline equivalence between experimental (*M* = 1.50, *SD* = 0.57) and control groups (*M* = 1.53, *SD* = 0.57), *t*(58) = −0.21, *p* = 0.839, 95% CI [−0.38, 0.32], with no significant differences in pretest cooperative behavior scores (Table 2).

**Table 2:** Homogeneity testing of cooperative behavior scores between groups.

Sample grouping	*n*	*M ± SD*	*t*	*p*	95% *CI*
Experimental group	30	1.50 ± 0.57	−0.205	0.839	[−0.38, 0.32]
Control group	30	1.53 ± 0.57			

### Intervention effects

Mauchly’s test indicated violation of sphericity (*W* = 0.768, *p* = 0.001), prompting Greenhouse–Geisser correction (*ε* = 0.812). A 2 × 3 mixed-design ANOVA with corrected degrees of freedom yielded: (1) Time main effect (*F*(1.62, 94.18) = 32.54, *p* < 0.001, partial *η*^2^ = 0.261). As detailed in Table 3, while the control group maintained stable scores across all timepoints (T1: *M* = 1.53, *SD* = 0.57; T3: *M* = 1.63, *SD* = 0.56), the experimental group demonstrated progressive improvement from baseline (T1: *M* = 1.50, *SD* = 0.57) to post-intervention (T3: *M* = 2.23*, SD* = 0.43). (2) Group × Time interaction (*F*(1.62, 94.18) = 18.80, *p* < 0.001, partial *η*^2^ = 0.177). The experimental group’s T1-T2 gain (+0.27 points) and T2-T3 gain (+0.46 points) (Table 3) significantly exceeded controls’ marginal changes (+0.07 and +0.03 points respectively).

**Table 3:** Descriptive statistics for cooperative behavior scores at three assessment points.

Group	Pre-test (T1)	Mid-test (T2)	Post-test (T3)
Experimental group	1.50 ± 0.57	1.77 ± 0.50**	2.23 ± 0.43***
Control group	1.53 ± 0.57	1.60 ± 0.56	1.63 ± 0.56

Within-group comparisons were adjusted using Bonferroni correction for multiple comparisons. **p < 0.01 for T1 vs. T2, ***p < 0.001 for T2 vs. T3. Effect sizes for significant differences are reported in the text.

Polynomial contrasts confirmed a dominant linear trend (*F*(1, 58) = 32.540, *p* < 0.001) with no quadratic component (*F*(1, 58) = 0.716, *p* = 0.401). *Post-hoc* tests with Bonferroni adjustment confirmed that the experimental group’s improvement from T1 to T3 represented a large effect (*d* = 1.32, 95% CI [0.82, 1.80]), with 81.3% of children achieving clinically meaningful cooperation levels (≥2 points) at termination compared to only 15.6% at baseline. Between-group comparisons at T3 showed the experimental group significantly outperformed controls (mean difference = 0.60, 95% CI [0.38, 0.82], *p* < 0.001), with a number needed to treat (NNT) of 4 to move one child from competitive to cooperative behavior categories. These effect sizes substantially exceed conventional thresholds for educational interventions, suggesting both statistical and practical significance of the staged music program.

### Data visualization

**Figure 2:**
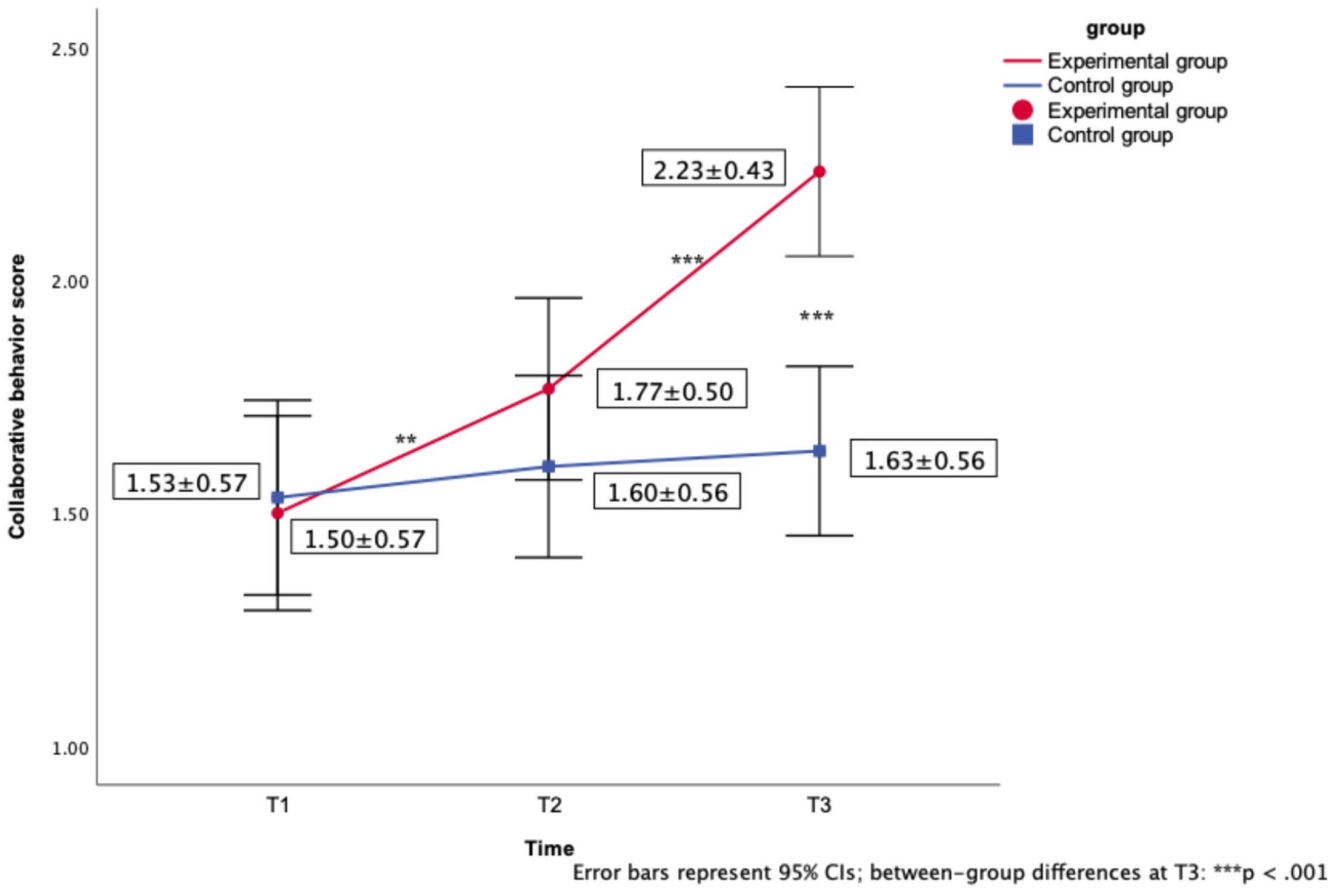
Changes in collaborative behavior scores between experimental and control groups across three time points.

As shown in Figure 2, the line graph clearly illustrates the experimental group’s progressive improvement (T1: 1.50 → T2: 1.77 → T3: 2.23) and the control group’s stable trajectory (T1: 1.53 → T2: 1.60 → T3: 1.63). Error bars (95% confidence intervals) indicate that the between-group difference peaked at T3.

### Robustness checks

Nonparametric Mann–Whitney U tests confirmed the experimental group’s superiority at T3 (*U* = 222.0, *p* < 0.001) despite heterogeneous variances (Levene’s test, *p* = 0.011). The large effect sizes (within-group: *d* = 1.32; interaction:*η*^2^ = 0.25) further validated the intervention’s robustness.

## Discussion

This study systematically examined the effects of action synchronization and instrumental ensemble playing on cooperative behavior in 5- to 6-year-old children through an 8-week phased group music game intervention. The main findings can be summarized in three aspects: (1) The action rhythm games in the first 4 weeks significantly improved cooperative behavior; (2) The instrumental ensemble games in the latter 4 weeks further reinforced the effects; (3) The intervention effects demonstrated temporal stability over short-term intervals, remaining significant in assessments conducted 1 week after each intervention phase. These results not only validate the theoretical hypothesis that musical synchrony promotes prosocial behavior (Kirschner and Tomasello, 2010) but also, through a phased design, provide the first empirical evidence of the sequential facilitation effect of motor synchronization and instrumental ensemble activities on cooperative behavior. This finding provides a new empirical perspective for understanding the relationship between musical activities and the development of prosocial behavior.

First, the phased intervention design aligns with developmental patterns. This study employed a two-phase intervention protocol progressing from motor synchronization to instrumental ensemble activities, reflecting the developmental trajectory of cooperative abilities from embodied interaction to symbolic collaboration. The first 4 weeks of the action synchronization phase involved rhythmic physical activities such as clapping and stepping, consistent with the cognitive development characteristic of “action preceding thought” in young children (Piaget, 1952). In the subsequent 4 weeks of the instrumental ensemble phase, children were required to translate rhythmic symbols into concrete actions, such as using percussion instruments to represent strong beats and string instruments to represent weak beats. This precise correspondence between symbols and actions helps promote the development of logical thinking. Additionally, real-time monitoring and adjustment of one’s own and peers’ performances during ensemble playing helps overcome egocentrism (Wan and Zhu, 2021). The research findings also align with Parten’s stages of play development theory (Parten, 1932), progressing from parallel play in the motor synchronization stage to cooperative play in the instrumental ensemble stage.

Second, gamified design stimulates children’s intrinsic motivation for cooperation (Perez-Aranda et al., 2023; Goagoses et al., 2023). The unique pleasure, interactivity, and immediacy of group music games effectively stimulate children’s motivation to cooperate. First, the pleasurable experience of music games provides an emotional driving force for cooperative behavior (Wu and Lu, 2021). Second, the interactive process enhances liking and trust among peers, reinforcing collective identity (Kara and Taner Derman, 2025; Good et al., 2017; Cirelli, 2018). Finally, the immediate feedback from the collaborative effect (e.g., rhythmic consistency) forms positive reinforcement, which may regulate prosocial behavior by influencing the neural activity of key brain regions in the reward system (nucleus accumbens, medial prefrontal cortex) (Walsh et al., 2023; Wang et al., 2021), prompting children to actively adjust their behavior to cooperate with others.

Third, structured process systems promote cooperative behavior. The intervention employs a four-step fixed process: Contextual introduction uses anthropomorphic stories to transform abstract cooperative tasks into concrete “helping” behaviors, stimulating children’s prosocial instincts and facilitating the formation of collective intent and joint attention (Etel and Slaughter, 2019); Imitation experiences ensure that all participants master basic skills, preventing cooperation withdrawal due to ability differences, while implicitly conveying cooperation rules through repeated practice, achieving the internalization of cooperation rules (Beck and Rieser, 2020); Group collaboration cultivates critical role awareness and perspective-taking abilities through role division and negotiation (Flavell et al., 1981); Comprehensive application promotes children’s development from mechanical coordination to flexible collaboration through integrated demonstrations and dynamic challenges. The above process aligns with children’s cognitive characteristics, progressively enhancing their cooperative abilities through motivation, skill-building, and practice reinforcement.

Fourth, neural mechanisms support the effectiveness of staged interventions. The intervention effects in this study may stem from neural-behavioral coupling triggered at different stages: the action synchronization stage primarily activates the mirror neuron system (Rizzolatti et al., 2001) and enhances interbrain synchronization (Cirelli et al., 2014), promoting joint attention and action empathy; while the instrumental ensemble stage significantly activates the prefrontal executive function network (Hyde et al., 2009), reinforcing social cognitive abilities such as rule adherence and intent understanding. This progressive activation from the sensorimotor network to the social brain network is consistent with (Kokal et al., 2011) multi-level model of interpersonal synchronization, explaining why the two-stage intervention produces sequential facilitation effects.

In summary, this study, by constructing a “bodily rhythm -instrumental ensemble” staged intervention model, empirically demonstrated for the first time in a natural educational setting the sequential facilitation effect of music synchronization training on cooperative behavior in 5- to 6-year-old children, resolving previous discrepancies regarding the effects of single music interventions and providing new perspectives for music intervention theory and practice.

Despite these important findings, the study has the following limitations: limited cultural diversity of the sample; lack of long-term follow-up data; the regular curriculum activities in which the control group participated did not involve structured cooperation comparable to that of the intervention group; and absence of neurophysiological measurement techniques. Future research is recommended to: (1) use an active control group (e.g., non-musical cooperative games); (2) explore potential mediator or moderator variables (e.g., children’s personality traits or family environment); (3) conduct cross-cultural comparisons; (4) extend follow-up periods; (5) refine age-differentiated studies; and (6) employ fNIRS or similar technologies to provide physiological evidence. These improvements will help further validate the promotional effects of group music games on cooperative behavior.

## Data Availability

The raw data supporting the conclusions of this article will be made available by the authors, without undue reservation.
